# The Energy Potential of Harvested Wood Fuel by Refugees in Northern Uganda

**DOI:** 10.1155/2022/1569960

**Published:** 2022-02-22

**Authors:** Bernard Barasa, Loy Turyabanawe, Gertrude Akello, Paul Makoba Gudoyi, Claire Nabatta, Andrew Mulabbi

**Affiliations:** Department of Geography, Kyambogo University, P.O. Box 1, Kyambogo-Kampala, Uganda

## Abstract

For the last three decades, Uganda has lost considerable natural vegetation cover in the refugee settlements and buffer zones due to the high demand for wood fuel and timber. It is worthy to note that the supplies of wood fuel are more likely to dwindle in the near future. This study explored the determinants of harvested wood-fuel choices and their energy potential. It also examined the implemented energy conservation measures and constraints faced by the refugees both in Palorinya and Imvepi refugee settlements in Northern Uganda. The data were collected by conducting household interviews and collection of wood species samples for energy potential laboratory analysis. Findings indicate that the major sources of wood fuel were firewood, charcoal, briquettes, and biomass fuels. The major refugee choices that determined wood-fuel collection included the family size of the house hold, culture, method of cooking, type of food cooked, high poverty levels, and availability of family labour (*P* ≤ 0.05). The sampled wood tree species had the highest energy potential were *Celtis durandii* (5,837 kcal/kg), *Parkinsonia aculeata* (5,771 kcal/kg), *Delonix regia* (5,153 kcal/kg), and *Bligihia unijugata* (5,034 kcal/kg). Access to wood fuel by the households was mainly constrained by limited household income levels, long distances trekked, and inadequate awareness about wood fuel sources and availability. To conserve wood fuel, the refugees deploy several measures including the use of mobile solar gadgets for cooking and lighting, taking up agroforestry, use of briquettes, adoption of energy-saving cooking stoves, and establishment of new woodlots. Therefore, to reverse this trend, the Ugandan government and development partners should prioritise energy investments by supporting cheaper energy alternatives such as mobile solar gadgets and energy-saving cooking technologies, and establishment of woodlots.

## 1. Introduction

Uganda is one of the countries in Africa, which hosts the highest number of refugees mainly from South Sudan, Democratic Republic of Congo, Rwanda, Burundi, Sudan, and Ethiopia among others who are settled in gazetted refugee settlements [1]. Over 1.4 million refugees are currently hosted in Uganda [2], excluding asylum seekers. Despite the current status of refugees and asylum seekers, the Ugandan Refugee Policy provides for the sheltering of refugees in either camps or settlements [3] and also provides for their free movement, access to land, and work [4]. In addition, the Refugee and Host Population Empowerment (ReHoPE) strategic framework calls for the improvement of the resilience of refugees and host communities to reduce reliance on donor aid [5]. The humanitarian contributions made by nongovernmental organisations, civil society groups, and host communities in helping refugees settle and have access to humanitarian aid and life support services such as livelihood empowerment programmes cannot be underestimated [6, 7].

There are currently eleven refugee settlements in Uganda, spread across the country to accommodate refugees coming from different directions. These are managed by camp commanders and staff from the United Nations High Commissioner of Refugees. However, in this study, only two refugee settlements of Imvepi and Palorinya were selected and investigated because of their degazettement status, nationality of refugees from South Sudan, and proximity to forest reserves and wetlands. The establishment of these refugee settlements has come at a cost to the Ugandan government leading to increased outbreak of human diseases from appalling sanitation and nutritional challenges [8, 9] and vegetation degradation [10, 11] that have stretched the nation's financial resources in treatment, surveillance, and enforcement of forestry policies. Most of the settlements are established on public land, which is near fragile ecosystems like forests, wetlands, grasslands, and water bodies across different regions. Vegetation cover in refugee settlements is highly degraded, primarily, because of the high demand for wood fuel used for cooking and heating [12]. Firewood is collected from within the settlements and host communities, while charcoal is mainly accessed from the host communities although some small quantities are also acquired from within the settlements [13]. Depending on the type of wood fuel utilised, the type of technology used to cook, as well as household size, the quantities harvested vary significantly [6]. The high craving by host communities for income through charcoal burning and sale of firewood escalates this condition further.

The continued influx of refugees in Uganda has made it difficult to reduce overharvesting of firewood and charcoal because most of the solutions to wood fuel crisis are implemented at a local or pilot scale but not at settlement or regional levels. This study also recognises that many studies that have investigated refugees and wood fuel have paid more attention to the technologies involved in the use of wood fuel [14, 15], access to wood fuel [16, 17], the impact of refugees on vegetation [18, 19], and wood fuel supply and demand [20–22]. However, little attention has been given to the determinant choices and constraints of wood fuel access, the energy potential of harvested firewood, and documentation of energy conservation measures implemented in the settlements. In contribution to this information gap, the objectives of this study were (i) to assess the determinants of wood fuel collection and use by refugees depending on household sizes (very small, small, medium, and large); (ii) examine the calorific potential of harvested firewood by the refugees; (iii) ascertain the constraints of wood fuel collection and use by the refugees; and (iv) to profile the energy conservation measures implemented in the refugee settlements.

**Figure 1:**
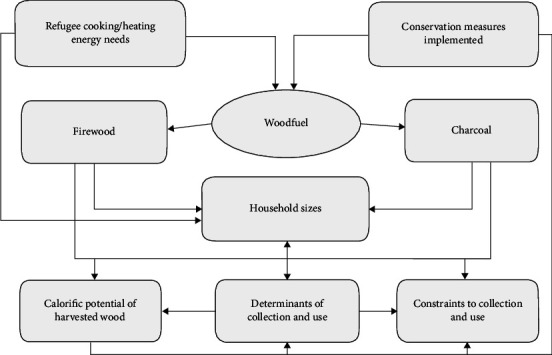
Conceptual framework of the study.

The study is conceptualised (Figure 1) around the refugees' cooking and heating energy needs that are met from available wood resources in form of wood fuel (firewood and charcoal). The need for firewood and charcoal depends on the refugee household sizes, and a number of factors determine collection and use under each household size category. The wood fuel used by refugees comes from different wood species with varying calorific potentials. However, some households are constrained from accessing fuelwood due to some challenges, and to ensure sustainability, stakeholders undertake some conservation measures.

## 2. Materials and Methods

### 2.1. Study Area

**Figure 2:**
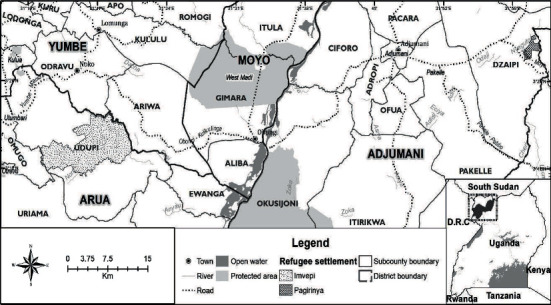
Location of the study area.

The study refugee settlements were Palorinya (37.58 sqkm) and Imvepi (52.937 sqkm) located in Northern Uganda (Figure 2). Palorinya is situated in Obongi District, while Imvepi is in Arua District. Palorinya settlement was established in 2016 (December), while Imvepi was set up in February 2017. The settlements lie within the shallowest part of the Albertine Rift, characterized by hills incised by deep valleys. Albert Nile forms the main surface drainage system. Soils in these areas are generally clay loams [23], and the native vegetation comprises mainly grassland, woodland, and bushland, although these have been converted to several land uses including settlement and agriculture.

The climate of the study area is tropical. Rainfall in the region follows a bimodal distribution pattern with lighter rains received between April and June and the second rains in August and October. The mean annual precipitation is 1,250 mm, whereas the average temperature ranges on the other hand between 20°C and 30°C [24]. The main economic activity in the study area includes crop growing especially maize, millet, and cassava. The primary sources of fuelwood are the woodlands, wetlands, and surrounding forests.

### 2.2. The Household Survey and Analysis

This study was field based, conducted using both qualitative and quantitative methods in 2019. The sampled population involved refugees and key informants from two refugee settlements (Palorinya and Imvepi). This study sampled 398 refugee households with a representation of 199 households from each. The sample size was determined following [25] sampling size determination procedures. This sample was sufficient to conduct in-depth key informant interviews, household face-to-face oral interviews, and focus group discussions. For the key informant and focus group discussions, interview guides were used, while at the household level, a semistructured questionnaire was used to investigate the determinants and constraints of wood fuel collection and use.

In each refugee settlement, a stratified random sampling method was used following the zonal camp refugee registration lists. Selection of refugee households was based on the daily use of wood fuel for cooking and residence time in the refugee settlement of at least a year. Data from the interviews were categorised according to household family size, i.e., very small (1–3), small (3–5), medium (6–8), large (9–12), and very large (12–15), to examine if these influenced the determinants of wood fuel collection and use. In addition, two focus group discussions consisting of 15 members (men, women, youths, elders, village leaders, and cultural leaders) were conducted in each settlement. Also, the key informants included settlement leaders, Officials of the office of the Prime Minister, United Nations High Commissioner for Refugees officials, District Natural Resources Officials (DNROs) from Obongi and Arua Districts, and members of the refugee-working group.

The significant determinants of wood fuel choices among the different household family size categories were tested using the binomial statistical test (BST), a nonparametric statistical test that compares responses to a variable with binary outcomes. The BST procedure compares the observed frequencies of the two categories of a dichotomous variable to the frequencies that are expected under a binomial distribution with a specified probability parameter [26].

### 2.3. Wood Fuel Sampling

Tree wood species samples were collected from each interviewed household. The woody biomass samples from the harvested tree species were collected, stored in cool and aerated containers, and transported to the laboratory for energy potential analysis. The species were classified according to weight, burning quality, moisture content, and size. Wood species weights were determined using mobile weighing scales, while burning quality and moisture content were determined in the laboratory. Samples were analyzed for dry matter using a vacuum oven at temperatures of 103°C, overnight until a constant weight was attained, while the energy values of woody species were determined using an oxygen bomb calorimeter.

## 3. Results

### 3.1. Sources of Wood Fuel Collection and Use

This study covered refugees from South Sudan hosted in Palorinya (Obongi District) and Imvepi (Arua District) refugee settlements located in Northern Uganda. The refugees revealed that they used relatively more firewood (84%) and charcoal (13%) followed by the use of briquettes (2%) and other biomass fuels (1%) such as agricultural residues, husks, and grasses for cooking. In the focus group discussions, the refugees reported having at least 2 meals per day and the wood fuel collection and production were largely carried out by children (especially the girl child) and women. The main sources of firewood included natural and artificial forests, bushes, and thickets. The respondents explained during the focus group discussions that this, however, does not come free as they must give part of their food ratios that include maize, beans, or cooking oil in exchange for firewood from the host community.

Firewood was mainly collected from the bushes, while charcoal was largely purchased from the host communities because the refugees are prohibited from burning charcoal within the settlements. However, the key informants reported that the nationals in the host communities have allowed the refugees to cut down trees in their land, from which charcoal is made but later share it in agreed proportions. The collections were largely made in the morning and evening times from either within the refugee settlements (30%) or the host community (70%). The majority of the refugees made at least 1–3 trips per week, while the rest made 5–7 trips, and the harvested firewood was largely transported on the heads and stored in kitchens, house verandas, and courtyards, while charcoal was kept indoors. The most frequently utilised technologies to cook are the three-stone fire (68%), clay charcoal stoves (27%), and rocket Lorena stoves (5%).

### 3.2. Composition of Refugee Families

The refugee families were composed of household heads, children, and relatives. Of the 389 participants, the majority of those interviewed were female household heads (71%). The interviewed respondents were largely married (75%), single (13%), widowed (6%), divorced (4%), and separated (2%) (Table 1). The average household age was 35 years. In terms of roles, the men were in charge of providing basic needs and engagement in productive activities like farming, while their female counterparts conducted reproductive roles, harvesting of firewood, and engagement in other productive duties. Insignificant livelihood options were largely derived from engagement in subsistence farming, causal labour, small-scale businesses, selling food rations, local brewing of alcohol, and formal employment. The children participated in firewood collection and charcoal purchase, collection of domestic water, cultivation, and livestock rearing. Relatives were made of orphans and immediate brothers and sisters of household heads. This union was within the refugee settlement and not at the source (country of origin). In terms of education levels, most of the participants had attained up to primary level, followed by those who had studied up to secondary level, while 12% had not attended any formal education. Only nine percent had reached vocational education level, while 3% had attained tertiary level education.

### 3.3. Determinants of Choices of Wood Fuel Collection and Use by Refugees in Relation to Household Size

The refuge household sizes had a significant contribution on the demand for wood fuel. Depending on the membership of each household, families were categorised as very small (1–3), small (3–5), medium (6–8), large (9–12), and very large (12–15) because these influenced the amount of food cooked and frequency. The household size categories that had a significant contribution to the collection and use of firewood were the large (9–12), medium (6–8), and very small (1–3) (*P* < 0.05) (Table 2). Other significant determinants were the size of the house, refugees' culture, weak enforcement of environmental laws and regulations, and availability of family labour (*P* < 0.05). This was followed by the methods used for cooking, high poverty rates, and the type of food cooked. Surprisingly, the household size categories that did not impact on the collection and use of firewood were the small (3–5) and very large (12–15) (*P* < 0.05). In the use of charcoal, the household categories of very small (1–3) and medium (6–8) opted more for charcoal followed by the small (3–5) category. In these categories, the determinants that did not significantly influence the uptake of charcoal were the availability of labour, type of food cooked, and culture (*P* > 0.05). However, the large (9–12) and very large (12–15) household size categories did not have any significant contribution on the use of charcoal by the refugees. One of the key informants revealed that intensive firewood collection, charcoal burning, and uncontrolled brick-laying activities were the leading causes of tree depletions.

### 3.4. Energy Potential of Harvested Firewood by Wood Species

**Figure 3:**
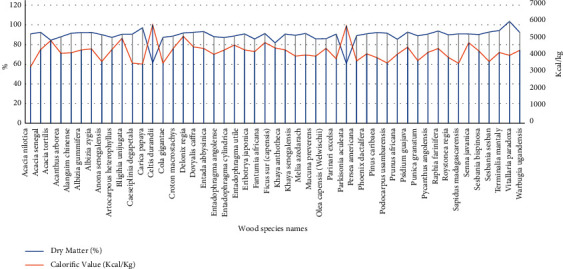
Calorific potential of harvested firewood by wood species.

Despite the categories of refugee household sizes, the collected head weights of firewood were made of various wood species with differing calorific potential (Figure 3). From the sampled firewood wood species, the frequency analysis showed that the most common varieties harvested were *Alangium chinense, Acanthus arborea, Funtumia africana,* and *Cola gigantea*. In terms of calorific potential, this study shows that the higher the percentage of firewood dry matter, the lower the calorific values, and vice versa. As per the wood density of collected firewood, the wood species with the highest calorific values were *Celtis durandii* (5,837 kcal/kg), *Parkinsonia aculeata* (5,771 kcal/kg), *Delonix regia* (5,153 kcal/kg), and *Bligihia unijugata* (5,034 kcal/kg). These wood species were common among the household size categories of large (9–12), medium (6–8), and very small (1–3) members.

### 3.5. Constraints of Wood Fuel Collection and Use by the Refugees

In the collection and use of firewood, the studied household size categories were mostly affected by low household income and long distances travelled in search of the wood (Table 3). This was followed by strict existing forest laws that hampered access to woody resources by the refugees unlike the household category of very small (1–3) household size, which attributed this state to limited awareness of the availability of firewood sources. Whereas in the acquisition and utilisation of charcoal, the household family size categories of very small (1–3) and medium (6–8) were affected by low household income and long distances. However, the small families (3–5) were principally controlled by strict forest laws and long distances, hence lesser encroachment. The large (9–12) household size category was constrained by indiscriminate cutting and wildfire caused by illegal seasonal bush burning practices. This finding further shows that the level of household income played a more critical role in the acquisition and use of firewood than charcoal.

### 3.6. Energy Conservation Measures Undertaken in the Refugee Settlements

**Figure 4:**
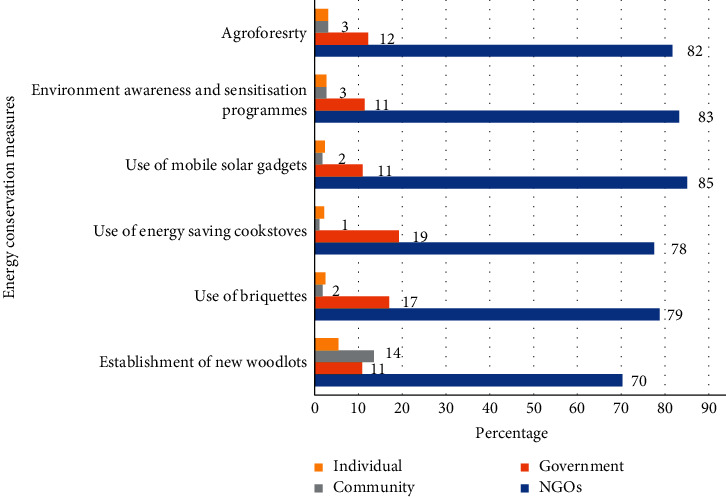
Wood fuel conservation measures implemented in the refugee settlements.

This result presents the wood fuel (firewood and charcoal) conservation measures implemented by the stakeholders in the refugee settlements (Figure 4). The measures are meant to minimise the degradation of natural vegetation cover, while the stakeholders in this context are the individual refugee households, community, government, and nongovernmental organisations (NGOs). To reduce the demand for the pressure on wood fuel, the stakeholders including refugees undertook conservation measures, such as the use of mobile solar gadgets for cooking and lighting, conducting environmental awareness and sensitisation programmes, and taking up agroforestry-related activities. This was followed by the use of briquettes, the adoption of energy-saving cooking stoves and the establishment of new woodlots. Our study reports that the provision of alternative sources of energy has the potential to reduce pressure on the demand for wood fuel by the refugees. The conservation measures were in response to the high demand for wood fuel arising from the increasing pressure from population of refugees in the settlements.

## 4. Discussion

### 4.1. Determinants of Choices of Firewood Collection and Charcoal Production by Household Size

Firewood is the main energy source for cooking and heating among the South Sudanese refugees settling in Northern Uganda [27]. For such a rural setting, traditional biomass becomes the most relied-on resource for various refugee household needs due to the economic conditions associated with humanitarian situations [14, 28–30]. On the other hand, a few refugee households use charcoal, which is accounted for by the fact that refugees are prohibited from burning charcoal by the host communities; thus the little that is used is obtained is bought by buying and exchanging for food ratios. Large family size had a significant influence on the determinants of wood fuel collection and use because it influences the type of food cooked and the frequency of cooking [28, 31]. This result relates to the view that per capita consumption of firewood decreases with increasing household size; an indication that large families are efficient users of wood fuel [32]. The availability of family labour was equally a significant determinant because wood harvesting is a labour-intensive activity. The more available the family labour, the more intensive the use of wood fuel. The household income level was also significant. Because of high poverty levels characterized by the refugees, many succumbed to the use of wood fuel [33]. Reference [30] found an inverse relationship between traditional biomass consumption and disposable income per capita, meaning that refugees, like other settlers, have higher chances of opting for other energy forms, but stick to the use of wood fuel due to low income. The size of the house was influential because the larger the house, the larger the storage space for wood fuel. A similar pattern is observed in the determinants of charcoal production and use. The findings also reveal that smaller families are more engaged in charcoal production irrespective of their income levels. Interventions aimed at encouraging refugees to diversify their income sources to curtail vegetation degradation are thus called for.

### 4.2. Calorific Potential of Harvested Firewood by Wood Species

The wood species with the highest calorific values were *Celtis durandii*, *Parkinsonia aculeata*, *Delonix regia*, and *Bligihia unijugata*. High calorific values are an indication of more lignin properties compared with cellulose because the former has a lower degree of oxidation that raises the heating value and thus higher calorific values [34]. Overall, the study sampled tree wood species with higher calorific values were softwood species, which are associated with higher carbon content and thus higher heating potential [34]. A similar study comparing hard- and softwood species further reported differences in calorific potential in the two categories [35]. The tree species with higher calorific values from the current study should be promoted in the woodlots allocated to refugees as well as other nontropical trees with similar potential, such as pine [36]. Some tree species with higher calorific values are also fast-growing woods [37], which makes them ideal for energy and environmental conservation in refugee situations.

### 4.3. Constraints to Firewood Harvesting and Charcoal Production by Refugees

Household income and distances travelled in search for wood affected wood fuel use, among the household size categories studied. These findings are related to observations from studies elsewhere [11, 28, 29, 32, 38–41]. Where the choices of fuel and adoption of improved stoves for cooking in biomass-dependent countries were influenced by collection costs, fuel prices, household income, and government policies [40, 41]. Whereas the rich have access to several sustainable energy options, the refugee households rely on wood fuel that can be accessed freely or cheaply. In Uganda, over-reliance on traditional biomass has been previously blamed on poverty [29], which has restricted usage of modern energy sources to less than 4 percent of the households. Refugees consequently face health- and food security-related risks due to restricted economic and education opportunities that even restrict access to alternative energy resources [42]. Strict forest laws, in the refugee settlements, are an indication of efforts to preserve the environment even when the humanitarian conditions make the task difficult.

### 4.4. Energy Conservation Measures Implemented by the Refugees

The main energy conservation measures adopted included agroforestry, use of mobile solar gadgets, use of energy-saving cook stoves, use of briquettes, and establishment of new woodlots. These are employed by individuals, refuge community, government, and NGOs. The study findings reveal low adoption of modern energy conservation measures, owing to the high poverty levels in refugee situations vis-à-vis high-cost requirements for modern energy conservation technologies, which resonance with findings by previous studies [15, 21, 29]. Unless the economic status of the refugees is improved through livelihood diversification, they will continue to rely on unsustainable wood fuel utilisation technologies that exacerbate forest degradation [43].

The study also notes that NGOs are the main stakeholders promoting energy conservation, followed by the government, while the community and individuals are meagrely involved. This is because refugees are mainly concerned with their immediate/short-term needs since they have lost hope to plan for the future [44]. Noted is the fact that some energy alternatives promoted by NGOs (mobile solar gadgets) and the government (energy-saving cook stoves) are acquired at a much higher cost and yet most refugees barely earn any income. Besides, use of mobile solar gadgets, which is the most widely promoted energy conservation measure, serves only a small fraction of the refugees' total energy requirements [15]. The alternatives to wood fuel presented to such communities need to therefore be more accessible, efficient, and reliable, lest the refugees will prefer the former energy sources [42].

Further revelation relates to the view that different stakeholders have prioritised different energy conservation strategies. The results reveal the absence of an integrated plan of implementation to increase the uptake of the various energy conservation measures. Community involvement is noted to be low, yet according to [45], any type of environmental programme or policy aimed at refugees is bound to fail if planning and implementation underscores contributions from the local host community. Interventions to promote energy conservation measures like the establishment of woodlots and agroforestry among refugees should bring on board all partners and ensure sensitivity to the likely conflicts to promote resilience and preparedness among vulnerable populations [46]. The establishment of woodlots in the study area was largely left in the hands of the community and individual refugee households. Whereas land for woodlots in the study area is allocated by the government, the refugees need to be supported to fully utilise the spaces for the intended purpose; otherwise, the economic motives are likely to overweigh the anticipated environmental benefits in the utilisation of such spaces.

## 5. Conclusions

The study area remains highly populated with refugee settlements from South Sudan, meaning that pressure on wood fuel is still far from ending unless this situation is overturned in the near future. This study reports forth that the refugees used more firewood followed by charcoal, briquettes, and other biomass fuels such as agricultural residues, husks, and grasses as cooking fuel. The household size categories that had a significant contribution to the collection and use of firewood were the large families. The most significant determinants were the size of the house, refugee culture, weak enforcement of environmental laws and regulations, and availability of family labour (*P* ≤ 0.05). In terms of calorific potential, this study reveals that the higher the percentage of firewood dry matter, the lower the calorific values, and vice versa. In firewood collection and use, the studied household size categories were mostly constrained by low household income and long distances travelled in search of wood fuel. To reduce pressure on the wood fuel, the stakeholders undertook conservation measures such as the use of mobile solar gadgets for cooking and lighting, conducting environmental awareness and sensitisation programmes, and taking up agroforestry-related activities. Our study reveals that the provision of alternative sources of energy has the potential to reduce overdependence on wood fuel by the South Sudan refugees in Uganda. Therefore, the government ought to support other cheaper energy alternatives like mobile solar gadgets and energy-saving cooking technologies, while the local administrators should integrate the refugee needs in the district development plans for equal distribution of resources at large. More sensitisation programmes of wood fuel conservation should be promoted by all stakeholders in the refugee settlements.

## Figures and Tables

**Table 1:** Sociodemographic characteristics of refugees sampled (*N* = 398).

Variables	Description	Statistics
Gender	Male	117 (29%)
	Female	281 (71%)
Marital status	Single	56 (13%)
	Married	299 (75%)
	Widowed	22 (6%)
	Divorced	14 (4%)
	Separated	7 (2%)
Education level	Primary	221 (56%)
	Secondary	118 (30%)
	No formal education	47 (12%)
	Vocational	9 (2%)
	Tertiary	3 (1%)
Source of income	Subsistence farming	212 (53%)
	Casual labour	93 (23%)
	Small-scale businesses	76 (19%)
	Selling ratio	9 (2%)
	Formal employment	5 (1%)
	Brewing alcohol	4 (1%)
Age	Mean	35
	Std. Deviation	13.5
	Minimum	13
	Maximum	83
Number of households	Mean	6
	Std. Deviation	3.6
	Minimum	1
	Maximum	48

**Table 2:** Determinants of wood fuel collection and use by refugee household sizes.

Determinants	Household size categories								Large (9–12)		Very large (12–15)	
	Very small (1–3)				Small (3–5)		Medium (6–8)					
	N	Observed prop.	Test prop.	*P* values	*N*	*P* values	*N*	*P* values	*N*	*P* values	*N*	*P* values
*Firewood collection and use*												
Household size	45	1	0.5		49		44		24	0.000^*∗∗*^	5	0.0620
Culture	21	1	0.5	0.000^*∗∗*^	29		25	0.000^*∗∗*^	13	0.002^*∗∗*^	4	0.1250
Poverty	48	1	0.5		83		71		23	0.000^*∗∗*^	6	0.0310^*∗∗*^
Weak enforcement	19	1	0.5	0.000^*∗∗*^	42		18	0.000^*∗∗*^	10	0.039^*∗∗*^	2	0.5000
Type of food cooked	17	1	0.5	0.000^*∗∗*^	61		41		15	0.000^*∗∗*^	5	0.0620
Method of cooking	5	1	0.5	0.062	18	0.000^*∗∗*^	35		10	0.002^*∗∗*^	2	0.5000
Availability of labour	5	1	0.5	0.062	18	0.000^*∗∗*^	18	0.000^*∗∗*^	6	0.031^*∗∗*^	2	0.5000
Size of the house	9	1	0.5	0.004^*∗∗*^	15	0.000^*∗∗*^	13	0.000^*∗∗*^	6	0.031^*∗∗*^		
High household income level	2	1	0.5	1.000	2	1.000	2	0.500	8	0.008^*∗∗*^		
Availability of wood	3	1	0.5		3		27		11	0.001^*∗∗*^		

*Charcoal collection and use*												
Household size	35	1	0.5		28		12	0.000^*∗∗*^	5	0.0620	2	0.500
Culture	19	1	0.5	0.000^*∗∗*^	12	0.000^*∗∗*^	5	0.062	3	0.2500		
Poverty	16	1	0.5	0.000^*∗∗*^	16	0.000^*∗∗*^	9	0.004^*∗∗*^	3	0.2500	2	0.500
Weak enforcement	17	1	0.5	0.000^*∗∗*^	13	0.000^*∗∗*^	7	0.016^*∗∗*^	3	0.2500		
Type of food cooked	20	1	0.5	0.000^*∗∗*^	34	1.000	9	0.004^*∗∗*^	3	0.2500		
Method of cooking	15	1	0.5	0.000^*∗∗*^	20	0.000^*∗∗*^	8	0.008^*∗∗*^	2	0.5000		
Availability of labour	4	1	0.5	0.375	8	0.109	1	0.021^*∗∗*^	2	0.5000		
Size of the household	11	1	0.5	0.001^*∗∗*^	12	0.000^*∗∗*^	7	0.016^*∗∗*^	2	0.5000		
High household income level	9	1	0.5	0.004^*∗∗*^	10	0.002^*∗∗*^	6	0.031^*∗∗*^	2	0.5000	4	0.125

^*∗∗*^Significant at *P* ≤ 0.05.

**Table 3:** Constraints in firewood and charcoal production.

Constraints	Household size categories									
	Very small (1–3)		Small (3–5)		Medium (6–8)		Large (9–12)		Very large (12–15)	
	*N*	%	*N*	%	*N*	%	*N*	%	*N*	%
*Firewood production*										
Limited funds	47	27.2	53	20	50	23	17	18	3	19
Long distances	43	24.9	56	21	51	23	24	26	4	25
Limited awareness	31	17.9	29	11	23	10	9	10	2	13
Wildfires	6	3.5	16	6	9	4	6	6	1	6
Indiscriminate cutting	8	4.6	35	13	14	6	7	8	1	6
Strict forest laws	25	14.5	36	14	29	13	10	11	4	25
Sexual violence	4	2.3	15	6	13	6	4	4	1	6
Presence of wild animals	8	4.6	19	7	20	9	4	4	0	0
Tribal conflicts	1	0.6	6	2	8	4	6	6	0	0
Land tenure	0	0.0	1	0	3	1	6	6	0	0

*Charcoal production*										
Limited funds	29	23.8	9	11	7	18	3	13	1	17
Long distances	27	22.1	18	22	9	23	4	17	1	17
Limited awareness	14	11.5	14	17	2	5	3	13	2	33
Wildfires	1	0.8	5	6	1	3	4	17	0	0
Indiscriminate cutting	17	13.9	8	10	7	18	6	25	0	0
Strict forest laws	18	14.8	17	20	6	15	1	4	0	0
Sexual violence	8	6.6	3	4	3	8	0	0	1	17
Presence of wild animals	3	2.5	5	6	4	10	1	4	0	0
Tribal conflicts	0	0.0	1	1	0	0	1	4	0	0
Land tenure	5	4.1	3	4	1	3	1	4	1	17

## Data Availability

The socioeconomic and wood species' calorific data used to support the findings of this study are available from the corresponding author upon request. The request should be made through Barasa Bernard, Department of Geography, Kyambogo University, P.O. Box 1, Kyambogo-Kampala, Uganda Mob: +256 701 712526/0789 682122.
